# Comparison of the biometric parameters in patients with high myopia and anisometropia

**DOI:** 10.1186/s12886-022-02450-7

**Published:** 2022-05-20

**Authors:** Jinkun Liu, Yuhong Wang, Weiyi Huang, Fei Wang, Yazhang Xu, Yingying Xue, Mengnan Wu, Fei Yu, Ruxin Gao

**Affiliations:** 1 Xiamen University Affiliated Xiamen Eye Center, Xiamen, China; 2 NHC Key Laboratory of Myopia, Fudan University, Shanghai, China; 3 Key Laboratory of Myopia, Chinese Academy of Medical Science, Fudan University, Shanghai, China; 4 Eye Institute of Xiamen University, Medical College of Xiamen University, Xiamen, China

**Keywords:** High myopia patients, Anisometropia, Anterior biometric parameters, Radius of the anterior and posterior lens capsules, Axial length

## Abstract

**Background:**

To compare biometric parameters, especially lens parameters, in patients with high myopia and anisometropia.

**Methods:**

Patients with spherical equivalent greater than -6D and at least one eye with an axial length greater than 26 mm and a difference in binocular axial length greater than 2 mm were included in this study. In each patient, the eye with a relatively shorter axial length was assigned to Group S, and the other eye was assigned to Group L. In patients whose binocular axial length difference was greater than 4 mm, the eye with the shorter axial length was assigned to Group S1 and the other eye was assigned to Group L1. In patients whose shorter eye axial was less than 26 mm, the eye with the shorter axial was assigned to Group S2 and the other eye was assigned to Group L2. Central corneal thickness, corneal curvature radius, axial length, anterior chamber depth, lens thickness, white-to-white corneal diameter and the radius of the anterior and posterior lens capsules were compared between Group S and Group L, Groups S1 and L1, and Groups S2 and L2.

**Results:**

Sixty-four people were enrolled in the study. There were 26 people with an axial length difference more than 4 mm (Group S1 and Group L1) and 34 patients with an axial length less than 26 mm (Group S2 and Group L2). No significant differences were found in any parameters except axial length between Group S and Group L, Groups S1 and L1, or Groups S2 and L2 (*p* > 0.05).

**Conclusions:**

The anterior parameters of patients with high myopia did not change with the axial length.

## Introduction

Myopia is one of the main causes of vision impairment and has become an important public health problem worldwide in recent years [1]. In 2010, uncorrected refractive error accounted for 53% of severe and moderate vision impairment, and the global economic burden related to myopia was estimated to be $244 billion in 2015 [2]. With the excessive use of digital products, the incidence of myopia has increased considerably each year. Myopia affects our work, study and daily life, especially for patients with high myopia, which will cause a variety of serious complications, such as cataract, lens dislocation, maculopathy, retinal detachment, and eventually leads to irreversible blindness [3–5]. Many scholars devote their work to the study of high myopia. These studies can not only help us understand the characteristics and pathogenesis of high myopia but can provide insights for doctors when performing operations on patients with high myopia [6–8]. High myopia usually occurs in both eyes and is symmetrical on both sides. However, there are a few patients whose axial lengths are significantly different; for some people, one eye is normal and the other eye has high myopia, or both eyes have high myopia and the condition is more serious in one eye. A comparative study of these patients can provide a better understanding of the biological characteristics and pathogenesis of high myopia and can avoid the influences of many factors, such as age, sex, environmental, individual and other factors. We have conducted an analysis of anterior segment characteristics in patients with high myopia and binocular axial lengths greater than 2 mm in our hospital since 2014. We aimed to further explore the pathogenesis of high myopia and the biological characteristics of the anterior segment and to provide insight into the clinical trials of secondary cataracts due to high myopia.

## Methods

This study is a prospective study. It followed the tenets of the Declaration of Helsinki and was approved by the ethics committee of the Xiamen Eye Center. Written consent providing permission to use the patient’s data was obtained from the patients or their parents.

Patients with spherical equivalent (SE) greater than -6D and at least one eye with an axial length greater than 26 mm and a difference in binocular axial length greater than 2 mm were enrolled in the study from April 2014 to September 2019. Patients who had other ocular diseases, such as corneal diseases, retinal detachment, choroid exudation, congenital glaucoma, and abnormal lenses, such as dislocation or subluxation, were excluded.

**Fig. 1:**
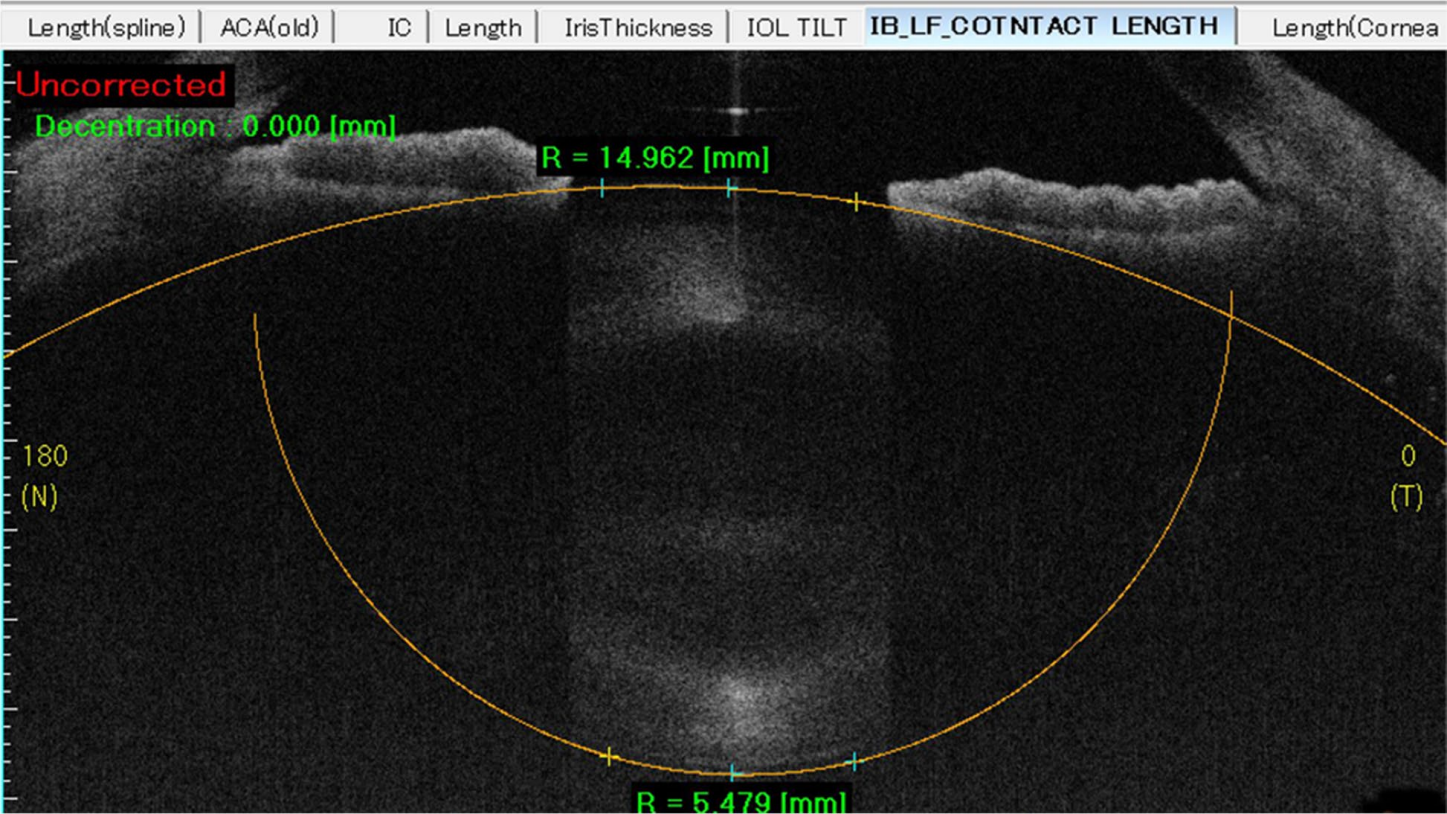
The radius of anterior and posterior curvature in the 4 mm central region of the crystal

Visual acuity, best corrected visual acuity, slit-lamp examination, fundus examination and medical optometry were performed for these patients. Intraocular pressure (IOP) was measured using the Goldman tonometer. Central corneal thickness (CCT), corneal curvature radius (CCR), axial length (AL), anterior chamber depth (from corneal rear surface to the front surface of the lens, ACD), lens thickness (LT) and white-to-white (WTW) corneal diameter were measured using a Lenstar 900 (HAAG-STREIT, USA). Ultrasound biomicroscopy (UBM) was performed to exclude lens abnormalities. Anterior optical coherence tomography (Tomey SS-1000 CASIA; Tomey, Nagoya, Japan) was used to measure the radius of the anterior and posterior curvature in the 4 mm central region of the crystal (radius of anterior capsule membrane, AR; radius of posterior capsule membrane, PR; Fig. 1). B scan, fundus photography, posterior OCT, and Opel examination were also performed to exclude other diseases.

The eye with a relatively shorter axial length of each patient was assigned to Group S, while the other eye with a longer axial length was assigned to Group L. CCT, CCR, AL, ACD, LT, WTW, AR and PR were compared between the two groups. Patients whose binocular axial length difference was greater than 4 mm were also analyzed. The eye with the lesser axial difference eye was assigned to Group S1, and the eye with the greater axial difference was assigned to Group L1. The above parameters were compared between both eyes. In addition, patients whose shorter eye axial was less than 26 mm were analyzed. The eye with the shorter axial was assigned to Group S2, and the eye with the longer axial was assigned to Group L2. The above parameters were also compared.

Statistical analysis was performed with SPSS software V.22 (SPSS, Inc., Chicago, IL). After identifying a normal distribution, a paired data t test was used for binocular comparison. *P* < 0.05 was considered statistically significant.

## Results

Sixty-four people (29 males, 35 females, aged 12 to 73 years) were enrolled in the study at Xiamen Eye Center. There were 26 people with an axial length greater than 4 mm (Groups S1 and L1) and 34 patients with a shorter axial length less than 26 mm (Groups S2 and L2). The AL in Group L is significantly larger than Group S (28.26 ± 2.04 mm VS 25.15 ± 2.08 mm), However, there were no significant differences in CCT, CCR, ACD, LT, WTW, AR and PR between the two groups (Table 1). When compared with Group L1 and Group S1, the difference of AL is greater than Group L and Group S, however, there is still no significant differences in CCT, CCR, ACD, LT, WTW, AR and PR (Table 2). The same results can be achieved when compared with Group L2 and Group S2 which has an AL shorter than 26 mm (Table 3).

**Table 1:** Comparison of bio-parameters between group S and group L

Parameters	Group S	Group L	*P*
AL (mm)	25.15 ± 2.08	28.26 ± 2.04	< 0.05
ACD (mm)	2.71 ± 0.36	2.99 ± 0.78	> 0.05
CCT (µm)	536 ± 39	531 ± 41	> 0.05
LT (mm)	4.12 ± 0.51	4.07 ± 0.53	> 0.05
CR (mm)	7.75 ± 0.33	7.8 ± 0.32	> 0.05
WTW (mm)	11.62 ± 0.85	11.52 ± 0.65	> 0.05
AR (mm)	15.54 ± 3.26	15.36 ± 3.38	> 0.05
PR (mm)	5.79 ± 0.17	5.76 ± 0.18	> 0.05

**Table 2:** Comparison of bio-parameters between group S1 and L1

Parameters	Group S1	Group L1	*P*
AL (mm)	24.01 ± 1.76	28.68 ± 1.72	< 0.05
ACD (mm)	2.87 ± 0.39	2.91 ± 0.57	> 0.05
CCT (µm)	520 ± 22	525 ± 22	> 0.05
LT (mm)	4.21 ± 0.53	4.18 ± 0.62	> 0.05
CR (mm)	7.45 ± 0.07	7.5 ± 0.14	> 0.05
WTW (mm)	11.45 ± 0.44	11.48 ± 0.48	> 0.05
AR (mm)	15.27 ± 3.12	15.19 ± 3.2	> 0.05
PR (mm)	5.68 ± 0.14	5.73 ± 0.15	> 0.05

**Table 3:** Comparison of bio-parameters between group S2 and L2

Parameters	Group S1	Group L1	*P*
AL (mm)	23.86 ± 1.1	27.22 ± 1.74	< 0.05
ACD (mm)	2.71 ± 0.37	3.14 ± 0.97	> 0.05
CCT (µm)	539 ± 27	535 ± 38	> 0.05
LT (mm)	4.0 ± 0.47	3.95 ± 0.45	> 0.05
CR (mm)	7.61 ± 0.24	7.66 ± 0.25	> 0.05
WTW (mm)	11.67 ± 0.48	11.57 ± 0.4	> 0.05
AR (mm)	15.78 ± 3.46	15.84 ± 3.36	> 0.05
PR (mm)	5.72 ± 0.15	5.74 ± 0.18	> 0.05

## Discussions

High myopia is increasingly becoming the leading eye disease that causes irreversible blindness in the world. Current research has mainly focused on the pathogenesis and changes in biological parameters, such as axial length, anterior chamber depth, corneal curvature, lens characteristics, and scleral, choroid and retinal changes. In our study, we aimed to determine the change in the anterior part of the eye through the comparison of parameters in patients with high myopia and anisometropia.

The anterior and posterior curvature radius of the lens is an important factor to describe crystal morphology. However, for the bulge of the iris in front of the lens, modern imaging equipment is unable to show the full picture and accurate biological parameters of the lens, lens thickness is the only parameter measured in clinical trials. The only published article about the curvature radius of the lens was conducted by Zheng in 2013, MRI was used to measure the change in curvature radius of the lens before and after adjustment in their article [9]. However, MRI examination cannot guarantee that the scanning plane is the central plane of the sagittal axis of the lens. In our study, anterior segment optical coherence tomography (AS-OCT) was used to accurately scan the lens image of the optic axis plane, and the anterior and posterior capsule images can be clearly viewed. The instrument's own software can measure the curvature radius of the anterior and posterior capsule of the lens in different diameter ranges semi-automatically and can also conduct 3D scanning imaging. In Zheng’s study, they found that the curvature radii of the anterior capsule and posterior capsule were 8.7 ± 0.8 mm and 6.2 ± 0.5 mm, respectively. As we all know, the radius of curvature of the cornea is generally 7–8 mm. The curvature radius of anterior capsule of the lens is slightly larger than that of the cornea in Zheng’s study and this is contrary to the fact that the anterior curvature of the lens is significantly larger than that of the front surface of the cornea from the OCT imagine. So, the results of Zheng’s study remain to be discussed.

The anterior chamber depth (ACD), central corneal thickness (CCT), corneal curvature and white-to-white distance were compared in patients with high myopia and anisometropia in our study. There were no significant differences between the two groups. This result is consistent with other studies [10–15]. However, contrary results in some parameters were found by other researchers [16–18]. The reason for the controversial conclusions of different scholars lies in the differences in research subjects, measuring instruments, and research designs.

In addition, we compared the biological parameters of patients with an axis length less than 26 mm and found that there were no significant differences in biological parameters other than axis length. Additionally, we drew the same conclusion when comparing patients with an axis length difference greater than 4 mm. So, we concluded that there is no change in anterior parameters, including the lens morphology, in patients with high myopia.

High myopia is a most common risk factor for advanced intracapsular IOL dislocation, with reported incidence ranging from 19.7 to 40% [19, 20]. Most researchers consider the suspensory ligament of patients with high myopia to be fragile and loose [19, 21, 22]. Hao Zhou discovered that the suspensory ligament is sparse and even reduced significantly in number in a guinea pig model of high myopia [23]. Na Liao found that the suspensory ligament length of patients with high myopia was significantly longer than that of patients with normal myopia (SD < -6.0 D) [24]. Other researchers consider that the lens capsule of patients with high myopia is larger and cause the excessive elongation of the zonular fibers for it has to support greater stress than in normal axial length eyes [19, 22]. The fragile and sparse zonular fibers causes the downward shift of IOL after cataract surgery [21]. Our research found that the lens thickness and curvature of the anterior and posterior capsules did not change as the axis of the eyeball lengthened. Therefore, we conclude that there is no change in lens morphology in patients with high myopia. The reason of the fragile and hyperextended suspensory ligament still needs more research.

## Conclusion

Our research compared the biometric parameters in patients with high myopia and anisometropia and concluded that there were no significant differences in ACD, CCT, corneal curvature, LT, anterior or posterior capsule curvature other than the axis length. This result is consistent with the theory that the pathogenesis of high myopia lies in the posterior part of the eye [25]. In addition, abnormalities in the suspensory ligament and vitreous cause lens instability in patients with high myopia, which leads to complications during and after cataract surgery. Although we compared the biometric parameters of patients with an axis length less than 26 mm and patients with a binocular axis difference greater than 4 mm, more detailed observation about the suspensory ligament is required in future research.

## Data Availability

The data analyzed during the current study are not publicly available due to the use of internal and confidential patient medical record data stored on internal, confidential and protected hard drives, but de-identified and redacted data are available from the corresponding author on reasonable request.
